# Additively Manufactured Geopolymer Monoliths as Robust Supports for High Temperature Catalytic Reactions

**DOI:** 10.1021/acsomega.5c09890

**Published:** 2026-01-03

**Authors:** Rafael Vidal Eleutério, Lisandro Simão, Maíra Palm, Rafael Catapan, Dachamir Hotza

**Affiliations:** † Graduate Program in Materials Science and Engineering (PGMAT), Federal University of Santa Catarina (UFSC), 88040-900 Florianópolis, Santa Catarina, Brazil; ‡ Research Group on Sustainability and Waste Management, Postgraduate Program in Environmental Technology, 42496 University of Ribeirão Preto (UNAERP), 14096-900 Ribeirão Preto, São Paulo, Brazil; § Graduate Program in Mechanical Science and Engineering (POSECM), Federal University of Santa Catarina, 89219-600 Joinville, Santa Catarina, Brazil; ∥ Graduate Program in Mechanical Engineering (POSMEC), Federal University of Santa Catarina, 88040-900 Florianópolis, Santa Catarina, Brazil; ⊥ Graduate Program in Chemical Engineering (POSENQ), Federal University of Santa Catarina (UFSC), 88040-900 Florianópolis, Santa Catarina, Brazil

## Abstract

Additively manufactured geopolymers are emerging as a versatile class of structured materials combining thermal and mechanical resilience for application in extreme environments. In this work, porous geopolymer monoliths were fabricated by material extrusion (MEX) using tailored metakaolin-based pastes, and printability was quantitatively assessed through a rheological protocol that links precursor attributes to processing behavior. The resulting 3D-printed structures (70% porosity, 30 m^2^/g) retained mechanical integrity after calcination at 800 °C, in contrast to conventionally cast counterparts that suffered severe strength loss. Upon Ni impregnation, temperature-programmed reduction (TPR-H_2_) evidenced strong metal–support interactions and the formation of stable nickel aluminosilicate phases, highlighting the role of the alkali rich geopolymer matrix in interfacial stabilization. As a proof of concept, methane steam reforming (MSR) was used to validate functionality under high temperature conditions (800-900 °C). Ni-geopolymer catalysts exhibited stable performance without deactivation over 2 h. These results position 3D-printed geopolymers as thermally stable, interface-engineered supports for high temperature catalytic technologies beyond the specific MSR case study.

## Graphical abstract

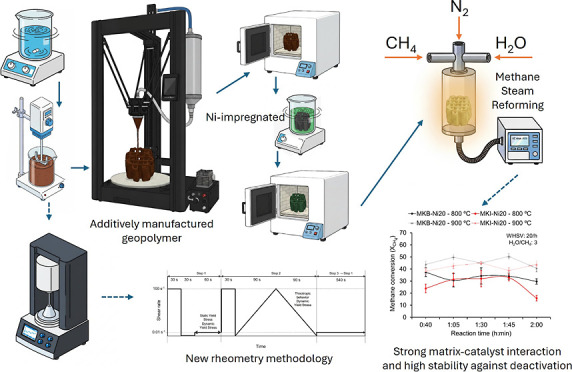

## 1 Introduction

The origins of geopolymer research as known today date back to the late 1950s, when Glukhovsky developed “soil cements” based on the alkali activation of clays. This work later inspired Davidovits,^1^ who in 1972 coined the term geopolymer to describe aluminosilicate materials synthesized using alkaline activators such as sodium or potassium silicates and hydroxides. The chemistry of alkali activated materials, however, has been explored since ancient times.^2^ These materials exhibit ceramic-like properties, high thermal stability, and chemical durability, making them attractive for various applications, including filtration,^3^ dye adsorption,^4^ and catalysis.^5^

Catalytic processes are central to the chemical industry, with heterogeneous catalysts widely employed due to their phase separation from reactants and products. Most catalytic systems use transition metals supported on high-surface-area materials capable of operation at elevated temperatures. Methane steam reforming (MSR), a key process for hydrogen production, operates above 700 °C and is representative of the severe conditions that challenge catalyst stability.^6,7^ Therefore, it is crucial to identify low-cost and thermally resistant support materials. Given their compositional tunability, ease of processing, and inherent thermal stability, geopolymers emerge as promising candidates in this context.

Additive manufacturing, also known as 3D-printing, particularly Material Extrusion (MEX), has enabled the fabrication of tailored geopolymer structures with hierarchical porosity and large surface areas.^8,9^ However, the printability of geopolymers depends strongly on their rheological properties, which are influenced by raw material composition and processing conditions.^10^ Despite efforts to define rheological parameters such as static/dynamic yield stress, viscosity, and open time,^6,11–16^ no standardized protocol for the evaluation of these parameters exists. The present work proposes a rheological testing method to better characterize geopolymer pastes formulated for MEX to address this issue.

In our most recent review on alkali-activated catalysts,^5^ we demonstrated that these materials offer several advantages for heterogeneous catalysis, including low cost, intrinsic alkalinity, and high thermal stability. Moreover, their ability to be synthesized from industrial wastes further increases their environmental appeal in a field traditionally reliant on energy-intensive raw materials and synthesis routes as cordierite or alumina. However, few studies have explored their application as catalytic supports in high-temperature processes. Existing reports in this context, such as the work of Candamano et al.,^14^ focused on powdered geopolymer-based materials in packed beds, without investigating additively manufactured structured monoliths. Other studies have examined structured geopolymer catalysts, though limited to low temperature applications such as biodiesel production^17^ and wastewater treatment.^11^ To the best of our knowledge, this is the first study to assess the suitability of structured alkali-activated materials for high-temperature catalytic applications, addressing a possibly important gap in the current literature.

In this paper, additively manufactured structured geopolymer monoliths produced via Material Extrusion (MEX) are evaluated as structured supports for Ni-based catalysts in high-temperature processes such as methane steam reforming (MSR), demonstrating that alkali-activated catalysts may represent a promising alternative to traditional ceramic and metallic supports due to their alkali-rich aluminosilicate framework, promoting strong metal–support interaction in an open porous structure capable of resisting sintered-induced deactivation. Rather than targeting catalytic performance optimization or providing detailed mechanisms of reaction, this study aims to assess the thermo-mechanical stability, physicochemical evolution, and functional integrity of the geopolymer framework under severe reaction conditions, thereby establishing a foundation for their strategic deployment in catalytic technologies.

## 2 Materials and Methods

### 2.1 Geopolymer Monolith Fabrication

#### 2.1.1 Raw Material Selection

Geopolymer monoliths were produced via extrusion of geopolymer pastes. Two commercially available metakaolin samples were used as aluminosilicate sources: Ultra HP (Metacaulim do Brasil), a gray powder named here as MK-B; and Metacaulim (Imerys), a white powder named as MK-I. Metakaolin is the most commonly employed aluminosilicate precursor in geopolymerization. The use of two different commercial sources enables assessing the influence of precursor characteristics on the ensuing geopolymer properties, as metakaolin reactivity is largely governed by its amorphous content, particle size, and chemical composition. For instance, the color differences are associated with compositional variations. Alkali activation was performed using a mixture of sodium silicate (Quimidrol, 10.8 wt % Na_2_O, 34.2 wt % SiO_2_, 55.0 wt % H_2_O) and a 10 M sodium hydroxide solution (Neon, 99 wt %). Only standardized, commercially available reagents were used to ensure reproducibility.

#### 2.1.2 Geopolymer Paste Preparation

The composition of the pastes employed was based on previous work^18^ to ensure adequate rheological properties for material extrusion (MEX). Polyethylene glycol (PEG-400, Neon, 98 wt %, MM = 400 g/mol) was used as a rheological modifier. Pastes were tested with PEG-400 at concentrations of 0%, 3%, and 5 wt %. A typical slurry, prepared for characterizations and extrusions, consisted of 40 g of metakaolin, 30 g of alkaline activators (1:1 wt. ratio of sodium silicate and sodium hydroxide), and 2.1 g of PEG-400 (3 wt %) as a rheological modifier. Samples prepared solely with MK-B were labeled MKB-0, while samples with a 25 wt % replacement of MK-B by MK-I were labeled MKI-0. The extruded geopolymer monoliths were designated MKB-G and MKI-G, derived from MKB-0 and MKI-0 pastes, respectively. The molar ratios for MKB-G were SiO_2_/Al_2_O_3_ = 3.25, Na_2_O/SiO_2_ = 0.19, Na_2_O/Al_2_O_3_ = 0.62, and H_2_O/Na_2_O = 12.22; for MKI-G, these values were 3.06, 0.19, 0.59, and 12.22, respectively. Sodium silicate was first added to the NaOH solution, and the alkaline activators along with PEG-400 were stirred for 10 min before being mixed with the metakaolin for 6 min. The resulting ink was then transferred to a 55 cm^3^ syringe.

#### 2.1.3 Catalytic Monolith Extrusion and Ni Impregnation

The pastes were extruded using a Duraprinter E-02 extrusion-based additive manufacturing device fitted with a 0.84 mm nozzle. G-code was generated via freeware software Slic3r from a CAD model of a cylindrical monolith (13 mm diameter and 11 mm height) with 21 layers and 30% infill in a rectilinear pattern, yielding a porosity of around 70%. PEG-400 (3 wt %) was added immediately before printing. The resulting geometry has 3 pores per centimeter (ppc) in the horizontal axis and 1.3 ppc in the vertical axis. The printing speed was set to 180 mm/s, and the samples were printed in room conditions over biaxially oriented polypropylene foils. To address the time-dependent rheological changes during printing due to polycondensation, extrusion pressure was manually adjusted (0.1-5 bar) to maintain a stable flow. Monoliths were printed under ambient conditions on biaxially oriented polypropylene sheets, cured at room temperature for 7 days, and washed with distilled water until electrical conductivity and pH stabilized.

Postcuring, the monoliths were calcined at 800 °C for 5 h (10 °C/min) to promote structural stabilization, remove residual organics and water. Nickel nitrate hexahydrate (Ni­(NO_3_)_2_·6H_2_O; Neon, 99 wt %) was used as the Ni precursor, and impregnation followed the methodology previously reported in the literature.^19–21^ In brief, the printed monoliths were immersed in an aqueous solution containing the calculated amount of precursor and gently heated to ∼100 °C until complete solvent evaporation. The required salt mass *m*_salt_ to achieve a target metal loading *m*_load_ (wt %) was calculated using eq 1, where *m*_sup_ is the mass of the support (g), and MM denotes the molar mass of the salt and nickel (g/mol). The calculated *m*_salt_ was multiplied by 1.5 to compensate for the absence of a washcoat layer, which increases precursor loss during impregnation due to reduced surface retention observed in preliminary tests. The impregnation was followed by a second calcination at 800 °C for 5 h (10 °C/min) to decompose the nitrate precursor into NiO and to further stabilize the catalyst structure.

$$m_{salt}=1.5\frac{m_{load}\cdot m_{\sup}\cdot MM_{salt}}{MM_{Ni}\cdot \left(100-m_{load}\right)}$$ 1

The efficiency of the evaporation-assisted impregnation is limited, and the effective *m*_load_ (wt %) was determined from the mass difference of the monoliths before and after impregnation. After impregnation, samples were calcined again at 800 °C (5 h) to decompose the nickel precursor and promote the formation of NiO onto the support surface.

### 2.2 Characterization Techniques

Table 1 presents a summary of the sample formulations used in this study, including their composition, physical form, and the characterization techniques employed for each material. Below, a brief description of each characterization procedure is provided.

**Table 1:** Summary of the Sample Formulations Used in This Study, Including Their Composition, Physical Form (Presentation), and the Characterization Techniques Employed for Each Material

sample	composition	presentation	characterization techniques
MK-B	metakaolin powder (metacaulim do Brasil)	powder	XRF, LOI, PSD, XRD, TGA/DSC
MK-I	metakaolin powder (Imerys)	powder	XRF, LOI, PSD, XRD, TGA/DSC
MKB-0	MK-B paste + PEG-400	paste	rheology, isothermal calorimetry
MKI-0	75% wt. MK-B paste + 25% wt. MK-I paste + PEG-400	paste	rheology, isothermal calorimetry
MKB-G	MKB-0 monolith	monolith	SEM-EDS, Porosity, BET, mechanical testing
MKI-G	MKI-0 monolith	monolith	SEM-EDS, Porosity, BET, mechanical testing
MKB-Ni20	MKB-G + 20% wt. Ni	monolith	SEM-EDS, BET, H_2_-TPR, strength, porosity/density
MKI-Ni20	MKI-G + 20% wt. Ni	monolith	SEM-EDS, BET, H_2_-TPR, strength, porosity/density

#### 2.2.1 Raw Material

The metakaolin powders (MK-B and MK-I) were characterized to assess their chemical, mineralogical, and thermal properties. Elemental composition was determined by X-ray fluorescence (XRF; EDX-7000), and the loss on ignition (LOI) was measured at 950 °C. Particle size distribution was obtained via laser diffraction (LUMiSizer, LUM GmbH) following ultrasonic dispersion to overcome particle agglomeration due to surface hydrophobicity. Mineralogical phases were identified by X-ray diffraction (XRD; Rigaku MiniFlex600) using Cu Kα radiation over a 2θ range of 5–80°, with a step size of 0.02° and a dwell time of 0.5 s per step. Diffractograms were interpreted using X’Pert Highscore Plus and Matchtbp software with the ICDD PDF-4 database. Simultaneous thermogravimetric analysis and differential scanning calorimetry (TGA/DSC) were conducted in a nitrogen atmosphere up to 1100 °C using a STA 449-F3 Jupiter (Netzsch) to investigate thermal stability and reaction events.

#### 2.2.2 Paste Characterization and New Rheological Testing Protocol

A custom rheological testing protocol was designed to simulate the material extrusion (MEX) process and evaluate early age thixotropy, static/dynamic yield stress, and viscosity evolution (Figure 1). Each test lasted up to 1 h or until the paste viscosity exceeded the rheometer’s operational limit. The protocol consisted of three sequential steps:

- Step 1: The paste was pre-sheared at 100 s^–1^ for 30 s to erase previous shear history, followed by a 30 s rest period, and then a constant shear rate of 0.01 s^–1^ for 60 s to determine static and dynamic yield stresses.
- Step 2: Used to evaluate the apparent plastic viscosity under a defined shear regime, leveraging changes in hardening behavior at early ages.
- Step 3: Simulated continuous extrusion and printing conditions, capturing rheological changes post-shear recovery.

**Figure 1:**
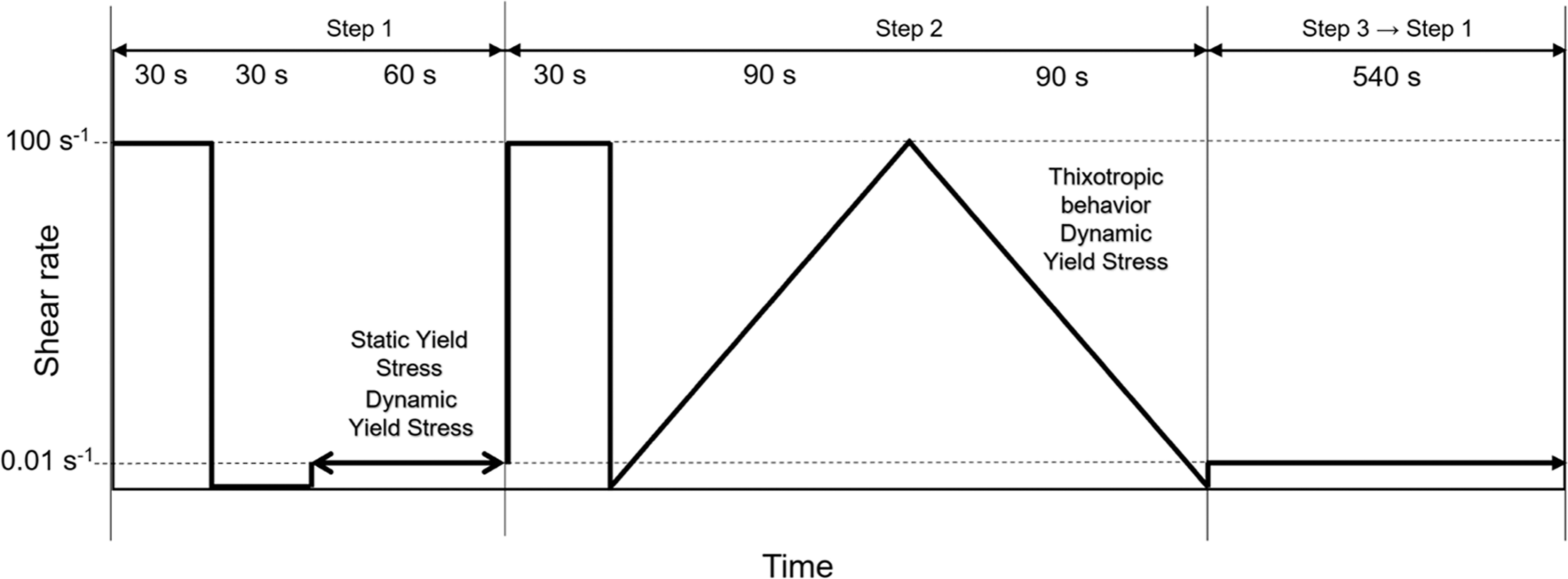
Rheology testing protocol.

Each formulation was tested in quintuplicate, 5 repetitions of the protocol without interruptions for the same evaluated paste, to assess reproducibility and robustness under conditions mimicking extrusion-based additive manufacturing. The protocol captures both irreversible rheopexy and reversible thixotropy typical of geopolymer systems during setting.

#### 2.2.3 Monolith Characterization

The morphology of the monoliths was analyzed via scanning electron microscopy coupled with energy-dispersive X-ray spectroscopy (SEM-EDS; Hitachi TM3030). Apparent porosity and bulk density were determined following ASTM C373,^22^ using the Archimedes method. True densities were measured using a gas pycnometer on ground samples (<75 μm). Geometric density was calculated from the measured dimensions and dry weights. Specific surface area was obtained from N_2_ adsorption measurements (single-point BET method) using a ChemBET Pulsar TPR/TPD Analyzer (Quantachrome Instruments). The thermal behavior of the samples was investigated via dilatometry (Expert System Solution Misura ODHT). The shrinkage of powdered geopolymer samples (MKB-G and MKI-G) was evaluated up to 1200 °C, while the thermal expansion of calcined monoliths (MKB-Ni0 and MKI-Ni0) was assessed up to 800 °C, with both tests conducted at a heating rate of 10 °C/min. Compressive strength was assessed according to ASTM C1231 ^23^ using a universal testing machine (Instron 5569) at a loading rate of 0.50 MPa/s. Hydrogen temperature-programmed reduction (H_2_-TPR) experiments were performed using the same ChemBET Pulsar system from ambient temperature to 1100 °C under a 5% H_2_/N_2_ flow (75 mL/min) at a heating rate of 10 °C/min to evaluate catalyst reducibility.

### 2.3 Evaluation of Catalytic Performance under Methane Steam Reforming

Methane steam reforming (MSR) experiments were conducted to evaluate the catalytic performance of the Ni-impregnated geopolymer monoliths (MKB-Ni20 and MKI-Ni20) at 800 and 900 °C, using a steam-to-carbon (S/C) molar ratio of 3:1. Reactions were carried out in a quartz tubular reactor (16 mm inner diameter, 473 mm length), placed inside a temperature-controlled furnace. The feed gas consisted of CH_4_ (99.99%), and N_2_ (99.999%) as a diluent. Water was introduced using a syringe pump into an evaporator, where it vaporized and mixed with N_2_. Gas flows were controlled via needle valves and measured by electronic flow meters. Weight hourly space velocity (WHSV) was set to 20 h^–1^, corresponding to a space velocity of approximately 17.8 NL/h·g_cat_. Estimated residence times were 58 ms at 800 °C and 53 ms at 900 °C. The parameters were set in intermediary values compiled by Meloni et al.^7^ for catalysts supported on ceramic carriers and according to the capability of the experimental setup. A schematic representation of the experimental setup is shown in Figure 2.

**Figure 2:**
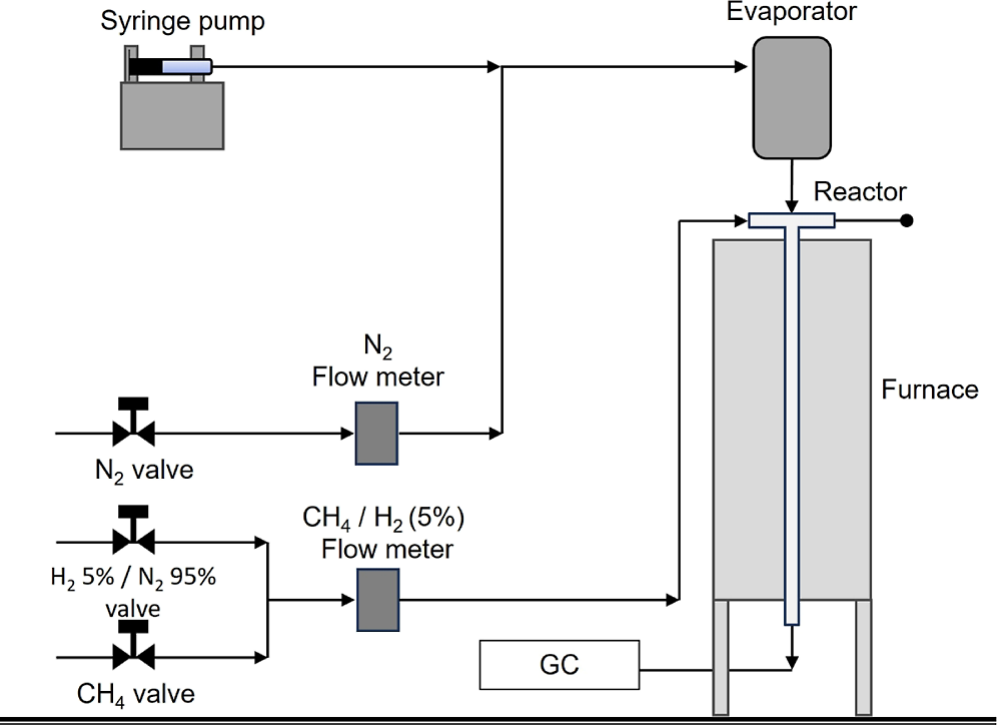
Schematic representation of a laboratory-scale MSR setup.

Before each run, catalyst reduction was performed in situ by heating the reactor to the reaction temperature at 10 °C/min under 100 mL/min of 5% H_2_/N_2_ (99.999%) and maintained for 1 h. The system was then purged with N_2_ and steam for 20 min before the reactant feed. Gas analysis was conducted using a Clarus 580 gas chromatograph (PerkinElmer) equipped with TCD and FID detectors and two capillary columns (Rt-U-BOND and Elite GC GS-Molesieve). Argon was used as the carrier gas, and calibration was performed with certified gas standards. After the run is completed, the catalyst was regenerated 100 mL/min of Class 0 air at 800 °C for 1 h to eliminate carbon deposition.

Methane conversion (XCH__4__) and hydrogen yield (*Y*_H_2__) were calculated according to

2 $$X_{CH_{4}}=\frac{{Ḟ}_{CH_{4},in}-{Ḟ}_{CH_{4},out}}{{Ḟ}_{CH_{4},in}}$$

3 $$Y_{H_{2}}=\frac{{Ḟ}_{H_{2},out}}{{Ḟ}_{CH_{4},out}+{Ḟ}_{H_{2},out}+{Ḟ}_{CO,out}+{Ḟ}_{CO_{2},out}}$$

where *Ḟ* stands for the molar flow rate. Typically, the calculated hydrogen yield (*Y*_H_2__) exhibits an estimated deviation up to 6%.

## 3 Results and Discussion

### 3.1 Raw Materials Characterization

The chemical compositions (XRF, wt %) of the metakaolin powders, MK-B and MK-I, are presented in the Table 2, and confirmed that both are high-purity aluminosilicates, with SiO_2_ and Al_2_O_3_ constituting over 92% of MK-B and 96% of MK-I by weight. MK-I exhibited higher purity, with lower concentrations of impurities such as Fe_2_O_3_ (0.85 wt %) and K_2_O (0.04 wt %) compared to MK-B.

**Table 2:** Chemical Composition (XRF, wt %) of MK-B and MK-I

oxides	MK-B	MK-I
SiO_2_	56.12	52.84
Al_2_O_3_	36.00	43.31
Fe_2_O_3_	2.27	0.85
TiO_2_	1.64	1.13
K_2_O	1.62	0.04
ZrO_2_	0.07	0.02
CaO	0.00	0.04
V_2_O_5_	0.06	0.00
SrO	0.02	0.01
MnO	0.02	0.00
CuO	0.01	0.01
Cr_2_O_3_	0.01	0.02
ZnO	0.01	0.00
NbO	0.01	0.01
PbO	0.01	0.01
Ga_2_O_3_	0.01	0.01
Y_2_O_3_	0.01	0.00
LOI	2.11	1.71

Mineralogical analysis by XRD (Figure 3a) revealed that MK-I possesses a highly amorphous structure, indicated by a prominent halo between 10° and 30° (2θ), which is characteristic of a reactive precursor for geopolymerization. In contrast, the MK-B sample was dominated by crystalline quartz peaks, with a less pronounced amorphous halo, suggesting lower intrinsic reactivity.^24^

**Figure 3:**
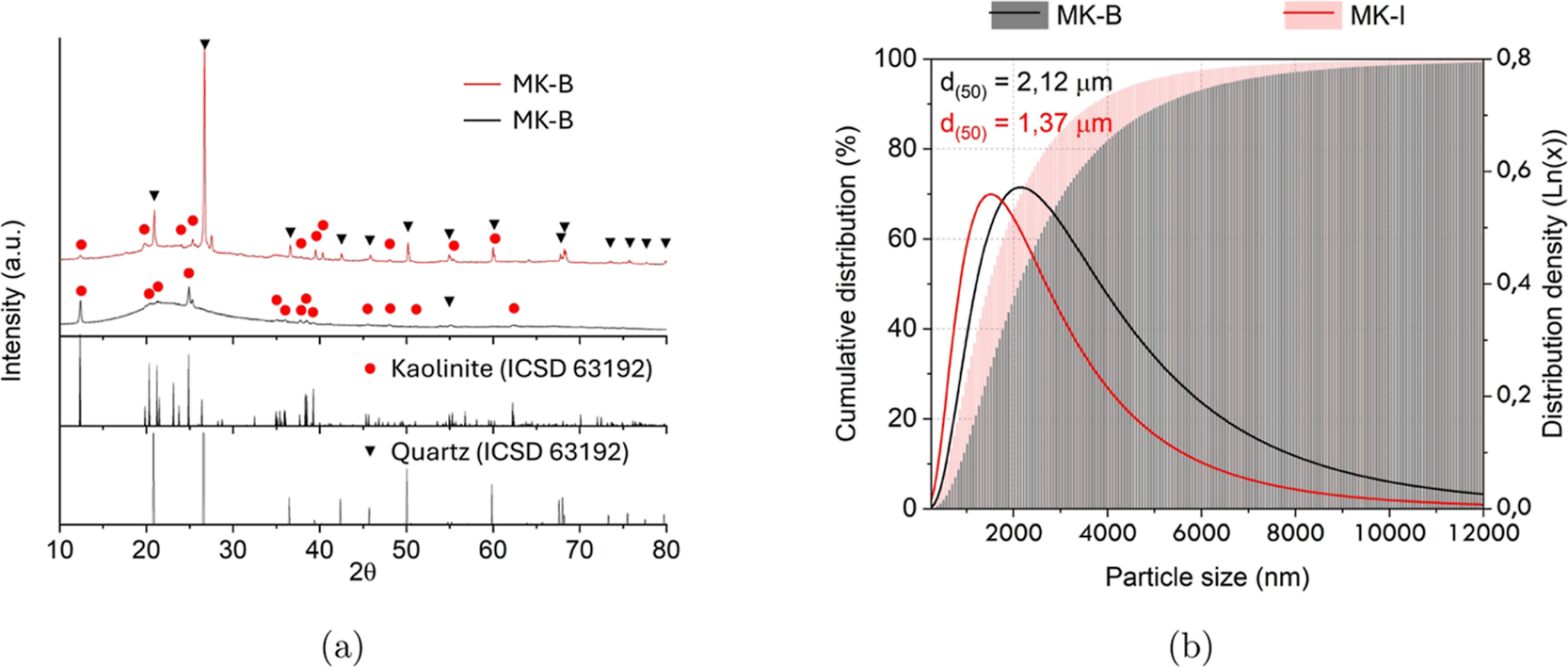
Characterization of MK-B and MK-I raw materials: (a) X-ray diffractogram (XRD) and (b) particle size and distribution.

The particle size analysis (Figure 3b) further distinguished the two materials, with MK-I having a smaller average particle size (*d*_50_ = 1.37 μm) and a narrower distribution compared to MK-B (*d*_50_ = 2.12 μm). These characteristics—higher purity, greater amorphization, and smaller particle size—indicated that MK-I would be significantly more reactive, leading to faster geopolymerization and higher paste viscosity. Preliminary extrusion tests confirmed this, as pastes formulated entirely with MK-I were unprintable; consequently, its content was limited to 25 wt % in the MKI-0 formulation to achieve rheological properties comparable to the MKB-0 paste.

Isothermal calorimetry was employed to evaluate the effect of the rheological modifier, PEG-400, on the reaction kinetics (Figure 4). The addition of PEG-400 effectively reduced the initial heat flow peak, thereby decreasing the geopolymerization rate and extending the paste’s workability window. A concentration of 3 wt % PEG-400 was found to be optimal, providing stable and reproducible extrusion, whereas 5 wt % resulted in an unstable process. The calorimetry results also showed that the MKI-0 paste had a higher reaction rate than MKB-0, as evidenced by a larger area under the heat flow curve within the initial 60 min.

**Figure 4:**
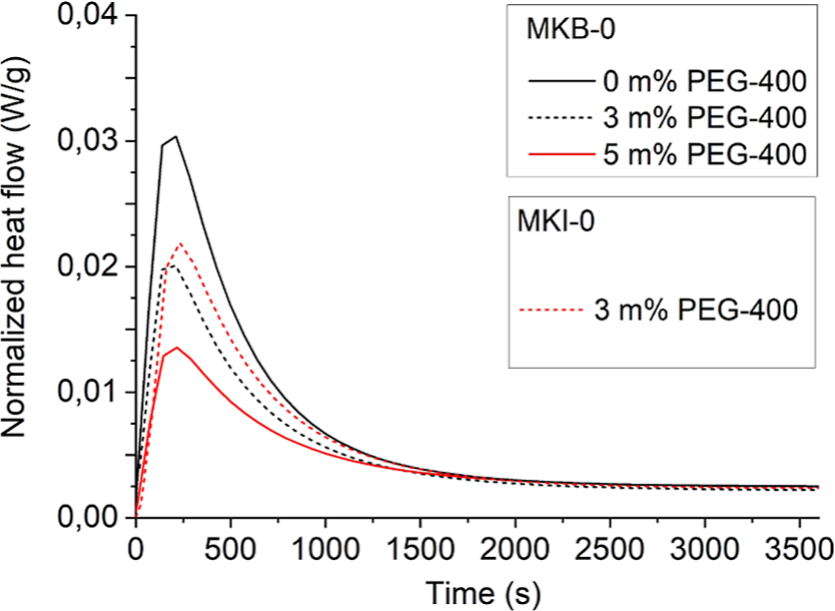
Isothermal calorimetry results for MKB-0 and MKI-0 pastes with PEG-400.

### 3.2 Rheological Behavior and Printability

For successful material extrusion (MEX) of geopolymer pastes, it is crucial to achieve the right rheological properties to ensure both printability and structural integrity. The paste must exhibit an adequate yield stress to prevent undesired flow during printing, while maintaining sufficient viscosity to support its shape after extrusion. Additionally, the paste should demonstrate shear-thinning behavior, where viscosity decreases under shear stress, facilitating smooth extrusion while ensuring rapid solidification after deposition. Thixotropy is another key factor, enabling the paste to recover its viscosity and resist deformation once extruded. Another essential parameter is the open-time window, which defines the period during which the paste maintains adequate buildability.^25^ These rheological properties directly influence the buildability of the paste and are governed by the concentrations of rheological modifiers, such as polyethylene glycol (PEG-400), and the composition of the geopolymer paste. The formulation should be optimized to suit the specific extrusion conditions, including nozzle size and printing speed.

The custom rheological protocol confirmed the distinct behaviors of the MKB-0 and MKI-0 pastes. As predicted by its material characteristics, the MKI-0 paste exhibited a higher initial static yield stress (approximately 627 Pa) compared to MKB-0 (480 Pa), attributed to its smaller particle size (Figure 5a). Over subsequent test runs, MKI-0 demonstrated a rapid and inconsistent increase in viscosity and yield stress, indicative of accelerated flocculation and gelation, which severely limited its open time for printing. In fact, the test for MKI-0 could not be completed beyond the fourth run as the paste’s viscosity exceeded the equipment’s limits. In contrast, the MKB-0 paste displayed a more stable and predictable rheological profile, with a gradual and consistent structural build-up over time, making it a more robust candidate for the material extrusion process.

**Figure 5:**
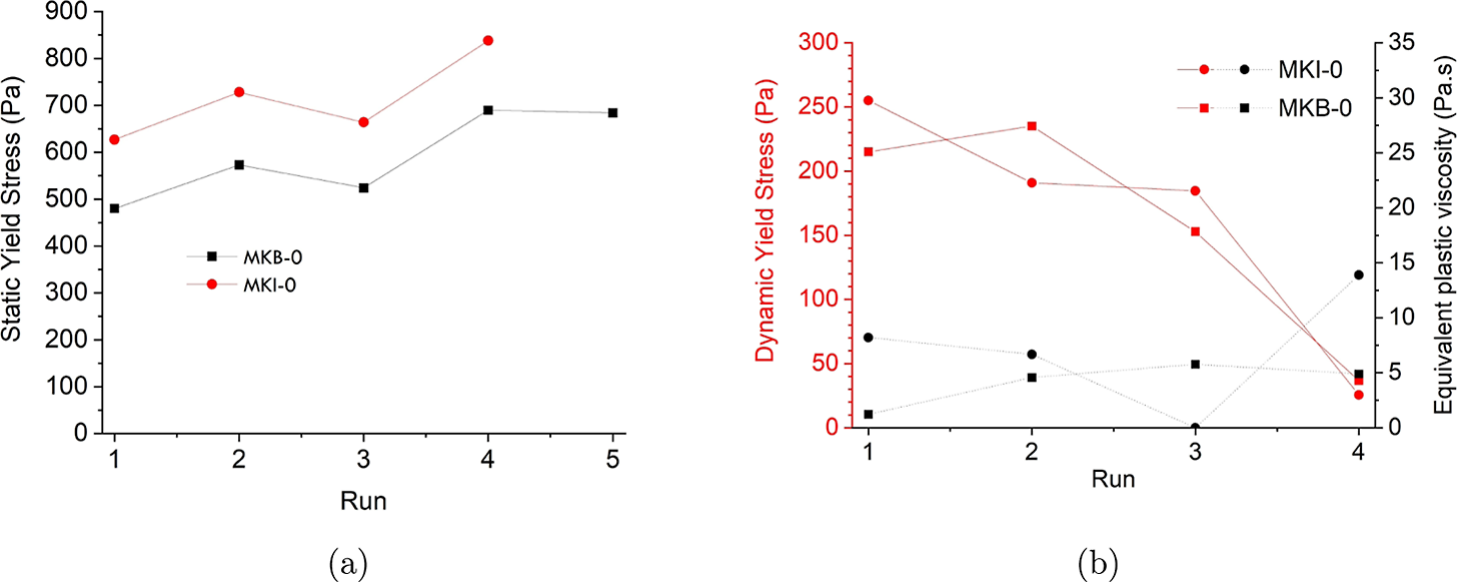
Rheological properties of MKI-0 and MKB-0 pastes: (a) Static yield stress evolution across multiple runs and (b) dynamic yield stress and equivalent plastic viscosity as a function of runs.

The dynamic yield stress for both pastes decreased after each high-shear step (Figure 5b), reflecting the thixotropic nature of the fresh geopolymers and the irreversible increase in viscosity when the material undergoes repeated shear–rest cycles (rheopexy). The findings underscore that while high reactivity is often desired in geopolymers, for additive manufacturing, a more controlled and slower reaction rate, such as that provided by the MK-B raw material, is crucial for achieving stable and reliable printability.

### 3.3 Monolith Characterization and Thermal Behavior

The additively manufactured monoliths, MKB-G and MKI-G, exhibited well-defined structures with excellent layer adhesion and strut integrity, as observed in SEM images (Figure 6). The final porosity of the monoliths (Table 3), 74% for MKB-G and 71% for MKI-G was slightly higher than the designed 70%, which was attributed to the intrinsic porosity of the geopolymer matrix itself, a feature visible in the microstructure. Notably, the specific surface areas of the printed monoliths were large (31 m^2^/g for MKB-G and 28 m^2^/g for MKI-G) for samples without using any porogenic fillers. These values are significantly greater than those reported for other additively manufactured geopolymers in the literature, which typically incorporate fillers and report surface areas in the range of 6–11 m^2^/g^17,26^ but lower than other unfoamed geopolymers.^27^

**Figure 6:**
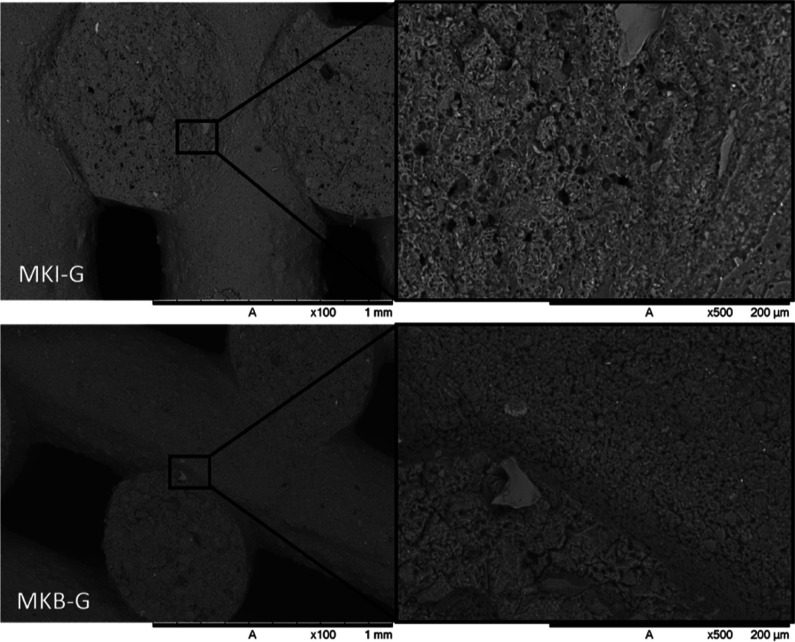
SEM micrographs of MKI-G and MKB-G monoliths at 100× magnification. Insets highlight the intrinsic porosity of the geopolymer matrix and the strut interfaces at 500× magnification.

**Table 3:** Fabricated Monoliths MKI-G and MKB-G: Specific Surface Area, Total Porosity, True Density, and Compressive Strength (Transposed View)

property	MKI-G	MKB-G
specific surface area (m^2^/g)	27.86	31.18
total porosity (%)	71 ± 3	74 ± 2
true density (g/cm^3^)	2.87	2.89
compressive strength (MPa)	20.9 ± 10.8	23.7 ± 12.2

The monoliths underwent significant physical changes after calcination at 800 °C for 5 h. The thermal treatment induces structural densification and partial crystallization of the geopolymer network, as it is typical for alkali-activated aluminosilicates exposed to high temperatures.^28^ This process is associated with the removal of structural water, migration of alkali species, and formation of thermally stable phases that contribute to the mechanical integrity observed after calcination, even though XRD patterns (Figures S1 and S3) did not present sufficiently defined peaks to conclusively confirm the formation of these stable phases.

As expected, shrinkage occurred, leading to a decrease in total porosity by 4–5% for both formulations and a substantial reduction in specific surface area to approximately 7 m^2^/g for MKB-Ni0 and 5 m^2^/g for MKI-Ni0. Despite this shrinkage, dilatometry tests showed that the calcined monoliths exhibited minimal expansion upon subsequent heating to 800 °C, confirming their excellent thermal stability for high-temperature applications (Figure 7).

**Figure 7:**
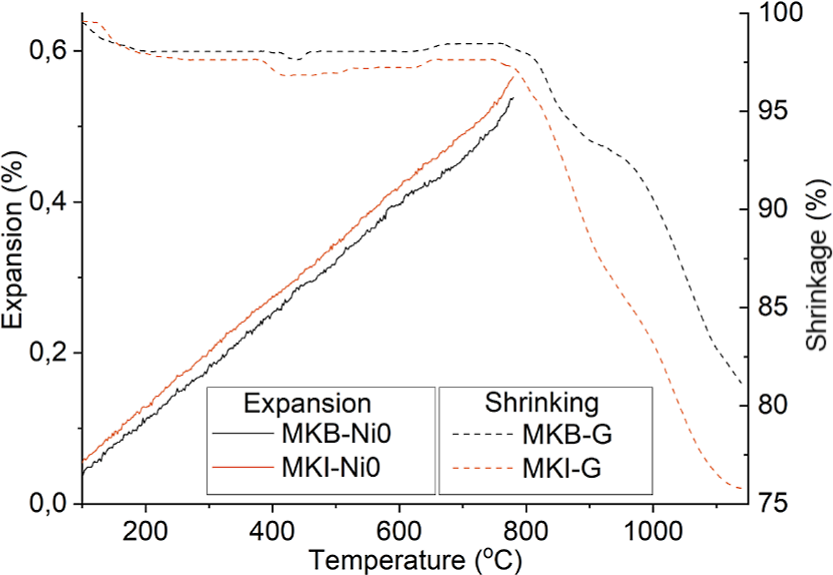
Thermal behavior of additively manufactured monoliths. After undergoing substantial shrinkage during the first calcination at 800 °C, the monoliths exhibited minimal dimensional changes when subsequently exposed to the same temperature, indicating thermal stabilization after the initial heat treatment.

A critical finding was the effect of thermal treatment on mechanical properties. While traditionally cast, dense cylindrical geopolymer samples experienced a drastic loss in compressive strength after calcination due to shrinkage and microcrack formation (Figure 8a), the additively manufactured porous monoliths did not (Figure 8b). The compressive strength of the printed samples remained high (e.g., 23.7 MPa for MKB) and was more dependent on the structural design and printing quality than on the thermal treatment. This demonstrates that the open-porous architecture effectively mitigates the internal stresses induced by thermal shrinkage, preserving mechanical integrity. This behavior may be associated with the crystallization observed by the XRD profiles (Figures S1 and S3) and densification that occurs during heat treatment, which tends to induce microcracking and weaken dense geopolymers, while its detrimental effects are attenuated in architected porous structures where stress is more efficiently dissipated.^28^

**Figure 8:**
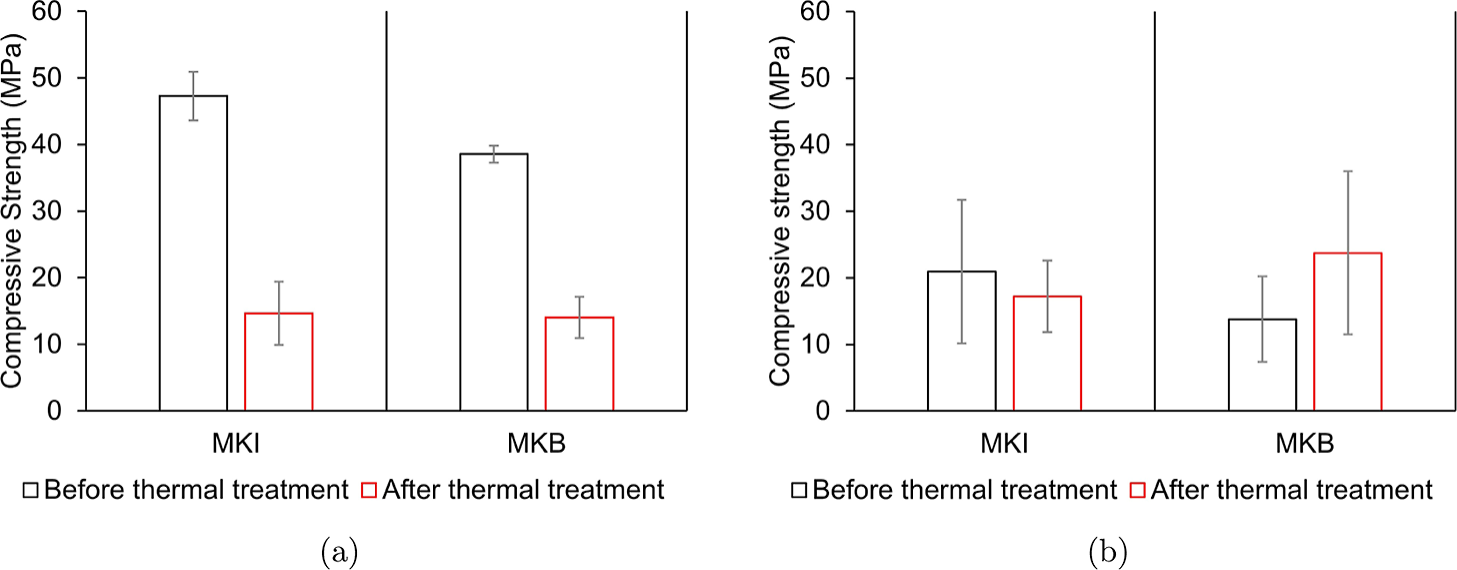
Compressive strength of (a) traditionally cast, solid cylindrical geopolymer samples and (b) compressive strength of material-extruded monolith samples before and after thermal treatment at 800 °C for 5 h.

### 3.4 Catalyst Characterization and Performance under Methane Steam Reforming

The geopolymer monoliths were successfully impregnated with nickel, achieving an average loading of 14 ± 3 wt % for MKB-Ni20 and 12 ± 3 wt % for MKI-Ni20 (Figure 9). SEM-EDS analysis confirmed a coating of nickel oxide on the monolith surface, which appeared as a cracked film, a common morphology for high metal loadings.

**Figure 9:**
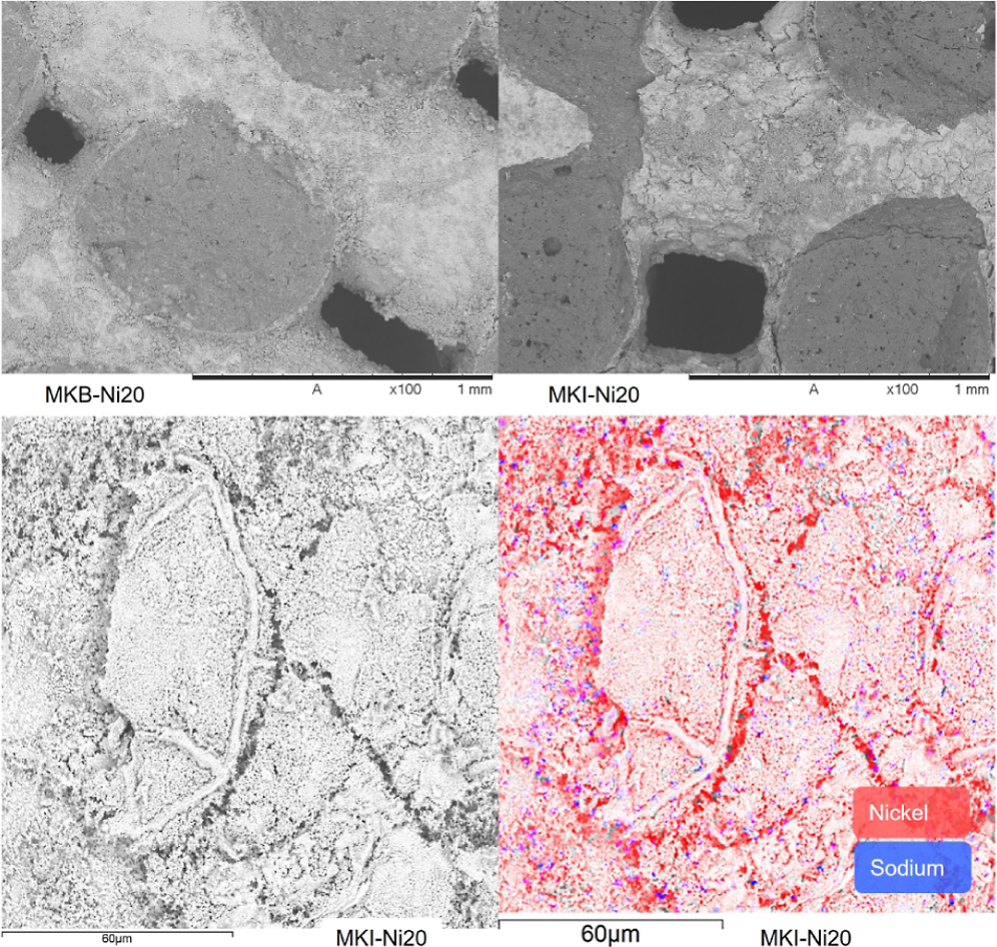
SEM images of MKB-Ni20 (upper left) and MKI-Ni20 (upper right) at magnification = 100×. The cracked film appearance is a common morphology for high metal loadings. Detail of MKI-Ni20 (bottom left) and elemental map by SEM-EDS (bottom right) showing the nickel coating on the material (red colored) and the presence of sodium (blue colored) on the surface.

Figure 10 provided insights into the metal–support interactions. The H_2_ consumption profiles for both MKB-Ni20 and MKI-Ni20 revealed a low-temperature peak around 400 °C, corresponding to the reduction of NiO with a weak interaction with the support. More importantly, multiple distinct reduction peaks were observed at higher temperatures (ranging from 500 °C to over 800 °C), which are indicative of strong metal–support interactions.^21,29^ These peaks strongly suggest the formation of complex, difficult-to-reduce nickel-aluminosilicate phases, such as a Ni_2_Al_2_SiO_8_ spinel, which were suspected but not definitively identified in the XRD profiles (Figures S2 and S4). The MKB-Ni20 sample showed greater total H_2_ consumption, which is consistent with its higher Ni loading and the presence of promoting elements like potassium in its raw material composition.

**Figure 10:**
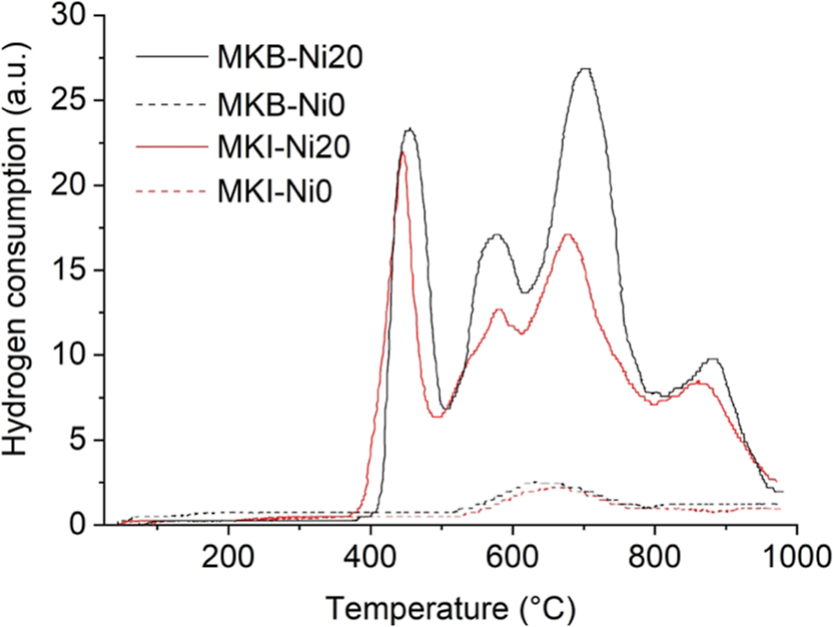
TPR-H_2_ profiles of additively manufactured geopolymer monoliths.

The thermal treatment at 800 °C induces structural densification and partial crystallization of the geopolymer network, as expected for alkali-activated aluminosilicates exposed to high temperatures.^28^ This process is associated with the removal of structural water, migration of alkali species, and formation of thermally stable phases that contribute to the mechanical integrity observed after calcination.

Strong metal–support interaction is a determining factor for the stability and lifetime of heterogeneous catalysts, particularly in high-temperature processes such as methane steam reforming, where active metal sintering and coke deposition are significant challenges. The literature consistently demonstrates that the presence of alkali and alkaline-earth metals in the support acts as a promoter, strengthening the interaction between nickel in the ceramic support.^29^ This effect results in enhanced resistance against Ni particle sintering and mitigates coke formation. The proposed mechanism suggests that these metals promote oxygen adsorption on the surface, which aids in the oxidation of intermediate carbon deposits. In this context, geopolymer supports offer an intrinsic advantage, as they are inherently rich in alkali metals, such as sodium, derived from the activators used in their synthesis.^5^ This composition contributes to the formation of the complex, thermally stable structures observed in the TPR-H_2_ analysis, which explains the robustness of the MKB-Ni20 and MKI-Ni20 catalysts during high-temperature operation.

In the methane steam reforming (MSR) evaluation, both geopolymer catalysts demonstrated stability over 2 h of reaction at 800 and 900 °C, with no signs of deactivation observed (Figure 11). As expected, methane conversion and hydrogen yield were higher at 900 °C for both catalysts. At 900 °C, the MKB-Ni20 catalyst exhibited superior performance, achieving a methane conversion of 45% and a hydrogen yield of 60%, compared to 40% and 55% for MKI-Ni20, respectively. This enhanced activity correlates directly with the characterization results, which showed MKB-Ni20 having a higher Ni content and stronger metal–support interactions.

**Figure 11:**
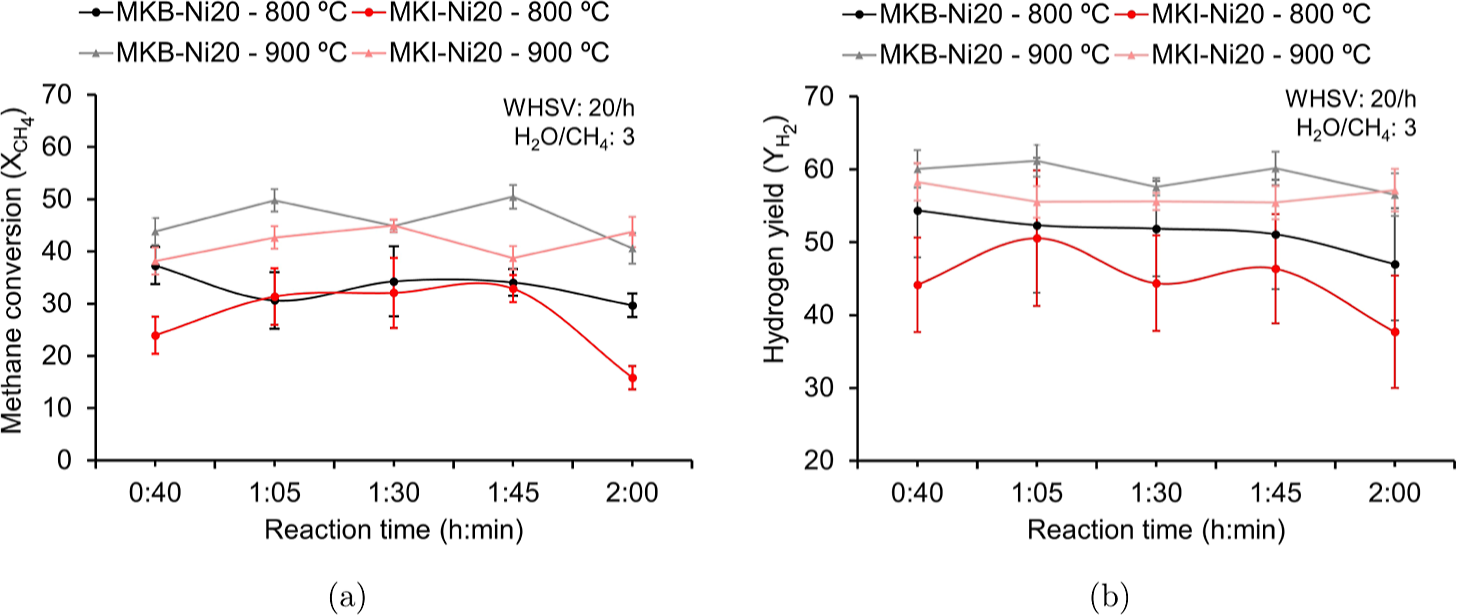
(a) Average methane conversion (*X*_CH_4__) and (b) average hydrogen yield (*Y*_H_2__).

Although the conversion values are lower than those of highly optimized commercial catalysts, the stability of the geopolymer support under these harsh conditions is a significant achievement. In contrast to reports where conventional Ni/cordierite catalysts show deactivation at lower temperatures,^30^ the Ni-geopolymer catalysts maintained stable performance, which is attributed to the robust SMSI preventing nickel sintering and coke formation. This was further confirmed by a regeneration experiment (Figure 12) where a spent MKI-Ni20 catalyst, after oxidating regeneration in air at 800 °C for 1 h inside the reactor, showed improved catalytic activity in a second MSR cycle at both. This suggests that the catalyst structure is stable and potentially enhanced by the thermal cycling, reassuring the positive and durable interaction between the nickel catalyst and the novel geopolymer support. The interconnected porous geometry facilitates gas diffusion and heat transfer throughout the catalyst body,^28^ while the sodium-containing aluminosilicate matrix promotes the formation of strongly interacting Ni phases. Together, these features contribute to the thermal stability and resistance to deactivation observed during MSR.

**Figure 12:**
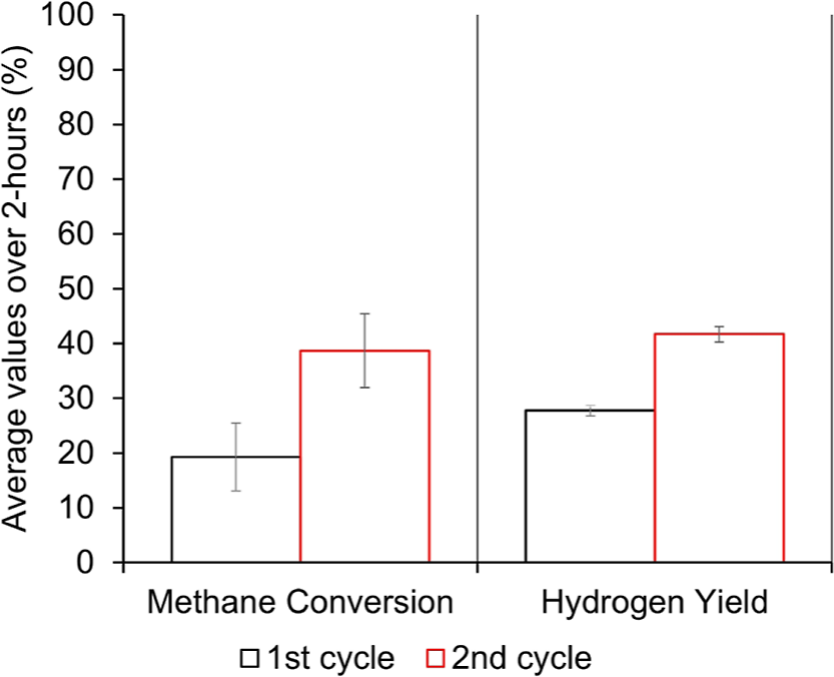
Average values of methane conversion and hydrogen yield in MSR for sample MKI2 for 2 h at 800 °C during the first cycle and after catalyst regeneration.

While the mechanistic aspects of methane steam reforming were not the focus of this study, it is widely established that MSR over Ni proceeds through methane dissociative activation on metallic nickel sites, followed by steam-assisted surface reactions that generate H_2_, CO, and CO_2_.^31^ The stable performance observed for the Ni–geopolymer monoliths is consistent with this mechanism, as the strong metal–support interaction promoted by the alkali-rich matrix likely contributes to maintaining nickel in a dispersed and active state under reaction conditions. Accordingly, the printed geopolymer framework appears to support the classical MSR pathway by preserving the accessibility and stability of Ni active sites at high temperatures.

## 4 Conclusions

This work demonstrates the strong potential of additively manufactured geopolymer monoliths as robust supports for nickel-based catalysts in high-temperature applications, particularly Methane Steam Reforming (MSR). By integrating raw material characterization, a tailored rheological protocol for MEX printability, and catalytic performance assessment under severe conditions, this work positions these materials as promising candidates for structured catalyst supports in high-temperature processes. Although no techno-economic or environmental assessment was conducted in this work, the potential for cost and environmental benefits derives from the fact that alkali-activated materials can be formulated using low-value industrial byproducts rather than energy-intensive raw materials, a point that future studies will explore. The main conclusions are summarized as follows:

- The printability of geopolymer pastes is critically dependent on the characteristics of the raw materials. Metakaolin with a larger average particle size and broader distribution (MK-B) provided superior workability and a longer open time for printing compared to the more reactive, finer, and purer metakaolin (MK-I).
- The newly proposed rheology protocol effectively characterized the structural build-up and thixotropic behavior of the pastes, proving to be a valuable tool for formulating geopolymer inks for material extrusion. The addition of 3 wt % PEG-400 was shown to be an effective method for reducing the initial geopolymerization rate, thereby extending the processability window.
- The additively manufactured monoliths achieved a high specific surface area of approximately 30 m^2^/g without using any fillers or porogenic agents. After calcination at 800 °C, the structures exhibited excellent thermal stability with low expansion, although the surface area was reduced to around 6 m^2^/g due to shrinkage.
- The porous, additively manufactured architecture was crucial for maintaining mechanical integrity after thermal treatment. Unlike traditionally cast solid samples, the printed monoliths did not lose compressive strength after calcination, a property attributed to the design’s ability to accommodate thermal stresses.
- Strong metal–support interaction between the nickel catalyst and the geopolymer support was confirmed through TPR-H_2_ analysis, which revealed the formation of highly stable, difficult-to-reduce nickel-aluminosilicate structures.
- The Ni-geopolymer catalysts demonstrated high stability and no deactivation during MSR at 800 and 900 °C for over 2 h. This robustness is attributed to the SMSI, which effectively prevents catalyst sintering and coking under harsh operating conditions. Furthermore, the catalyst proved to be regenerable, showing improved performance after a full reaction and regeneration cycle.

This work establishes a proof-of-concept for a new class of structured monoliths for high-temperature catalytic applications. Future efforts should prioritize monolith design optimization, the use of waste-derived precursors and their environmental impact, life-cycle assessment, improved catalyst dispersion, and the incorporation of thermal-conductivity-enhancing fillers to further enhance performance and expand the applicability of structured alkali activated materials in high-temperature catalysis.

## Supplementary Material



## Notes

XRD patterns of the samples investigated in this work (PDF)
