# Supplementary material for: Additively Manufactured Geopolymer Monoliths as Robust Supports for High Temperature Catalytic Reactions
Source: ACS Omega. 2026 Jan 3;11(2):3174–83. doi: 10.1021/acsomega.5c09890 (PMC12824935; doi:10.1021/acsomega.5c09890)
Supplement: Supplementary file 1 [file ao5c09890_si_001.pdf]

Supporting Information on the Paper: Additively manufactured geopolymer monoliths as robust supports for high temperature catalytic reactions

Rafael Vidal Eleutério,[†] Lisandro Simão,[‡] Maíra Palm,[¶] Rafael Catapan,[*,§,¶] and Dachamir Hotza[†,∥]

†*Graduate Program in Materials Science and Engineering (PGMAT), Federal University of Santa Catarina (UFSC), 88040-900 Florianópolis (SC), Brazil*

‡*Research Group on Sustainability and Waste Management, Postgraduate Program in Environmental Technology, University of Ribeirão Preto (UNAERP), 14096-900, Ribeirão Preto, SP, Brazil*

¶*Graduate Program in Mechanical Science and Engineering (POSECM), Federal University of Santa Catarina, 89219-600, Joinville, SC, Brazil*

§*Graduate Program in Mechanical Engineering (POSMEC), Federal University of Santa Catarina, 88040-900, Florianópolis, SC, Brazil*

∥*Graduate Program in Chemical Engineering (POSENQ), Federal University of Santa Catarina (UFSC), 88040-900, Florianópolis (SC), Brazil*

E-mail: rafael.catapan@ufsc.br

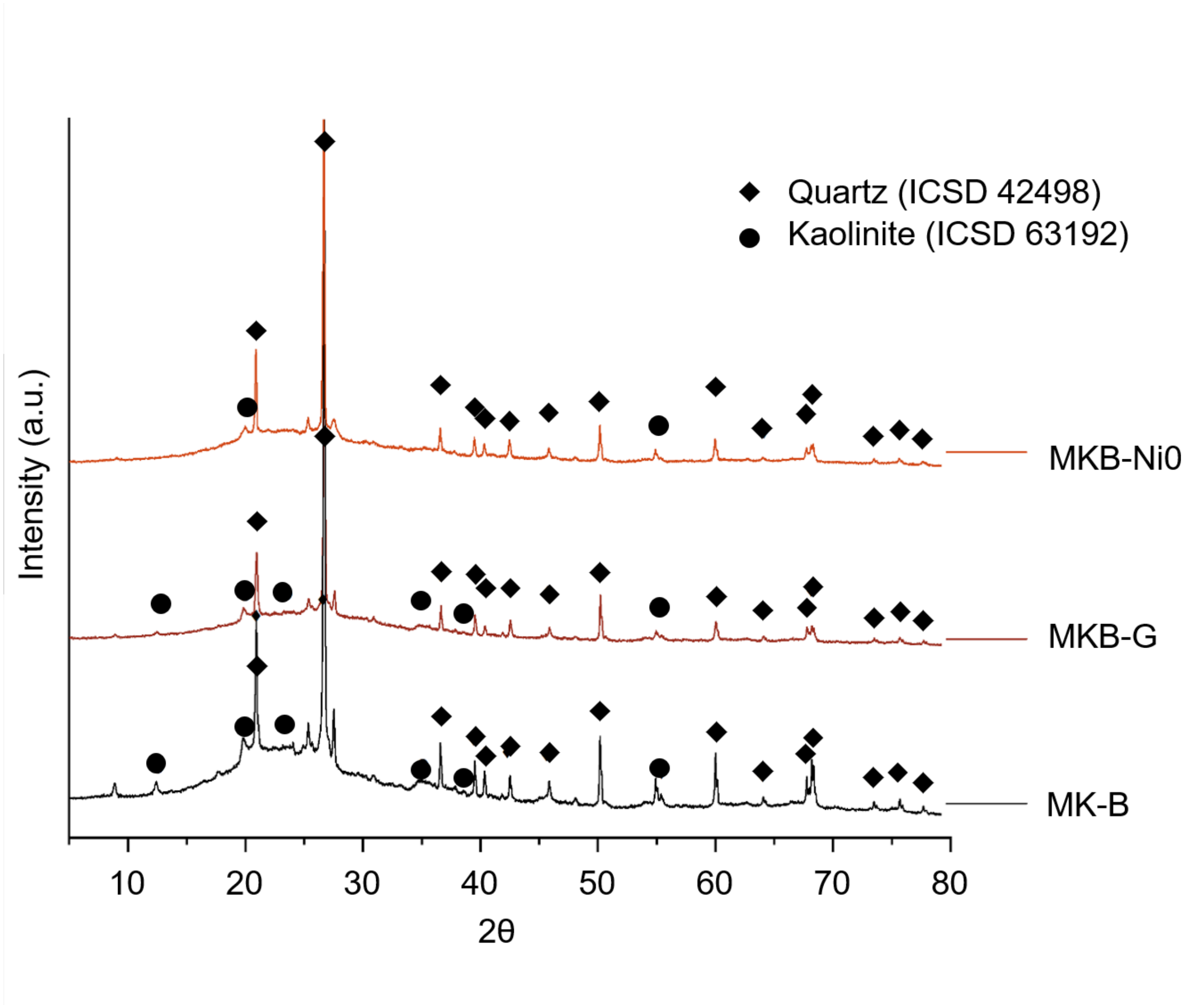

Figure S1: XRD profiles of MK-B (metakaolin), MKB-G (MK-B monolith) and MKB-Ni0 (800 °C calcinated MK-B monolith

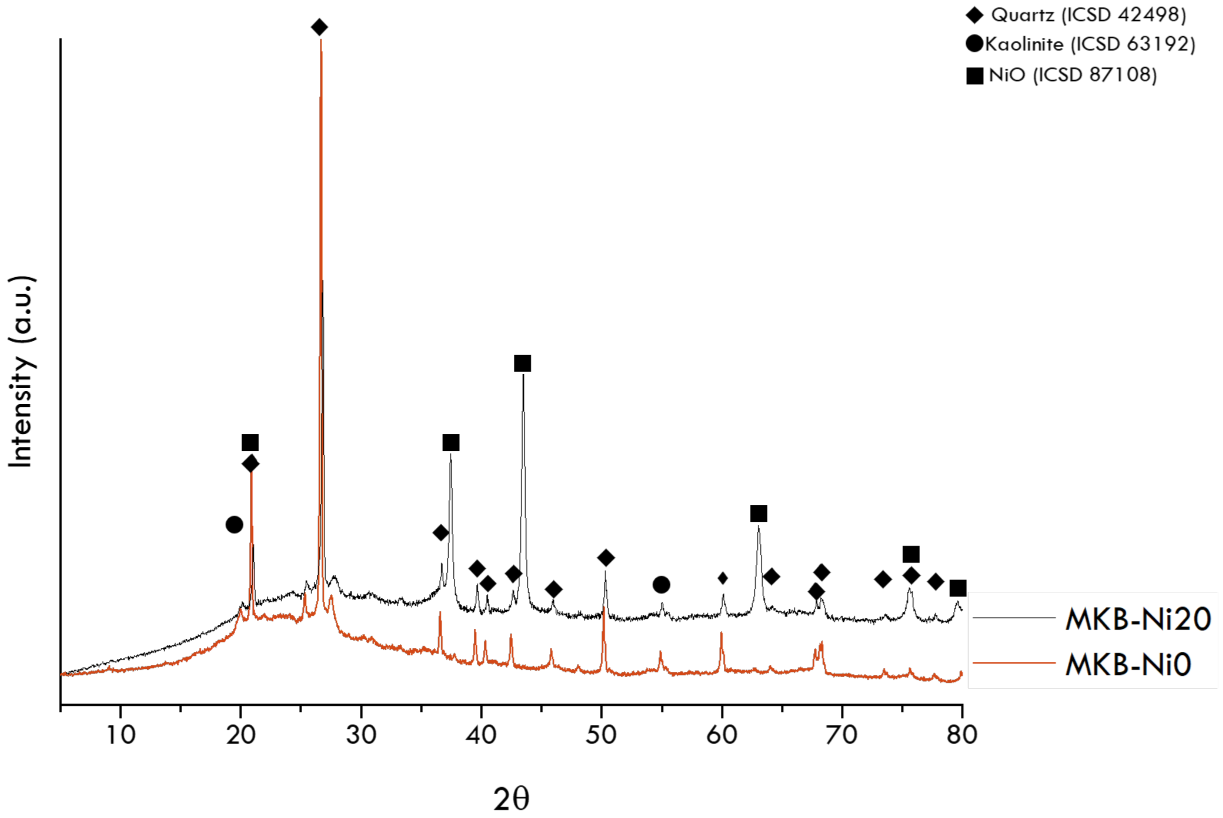

Figure S2: XRD profiles of MKB-Ni0 (800 °C calcined MK-B monolith) and MKB-Ni20 (800 °C calcinated Ni-impregnated MKB-Ni0 monolith.

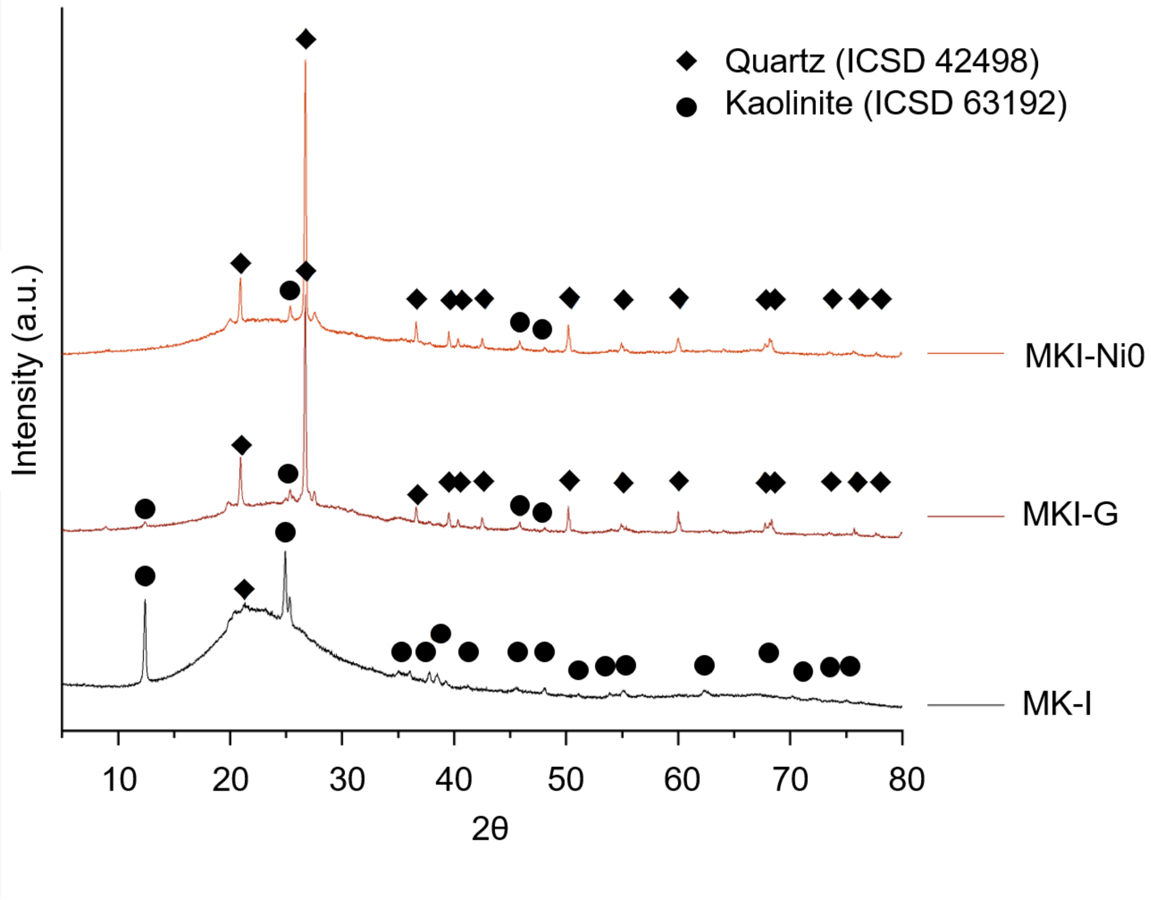

Figure S3: XRD profiles of MK-I (metakaolin), MKI-G (MK-I monolith) and MKI-Ni0 (800 °C calcinated MK-I monolith.

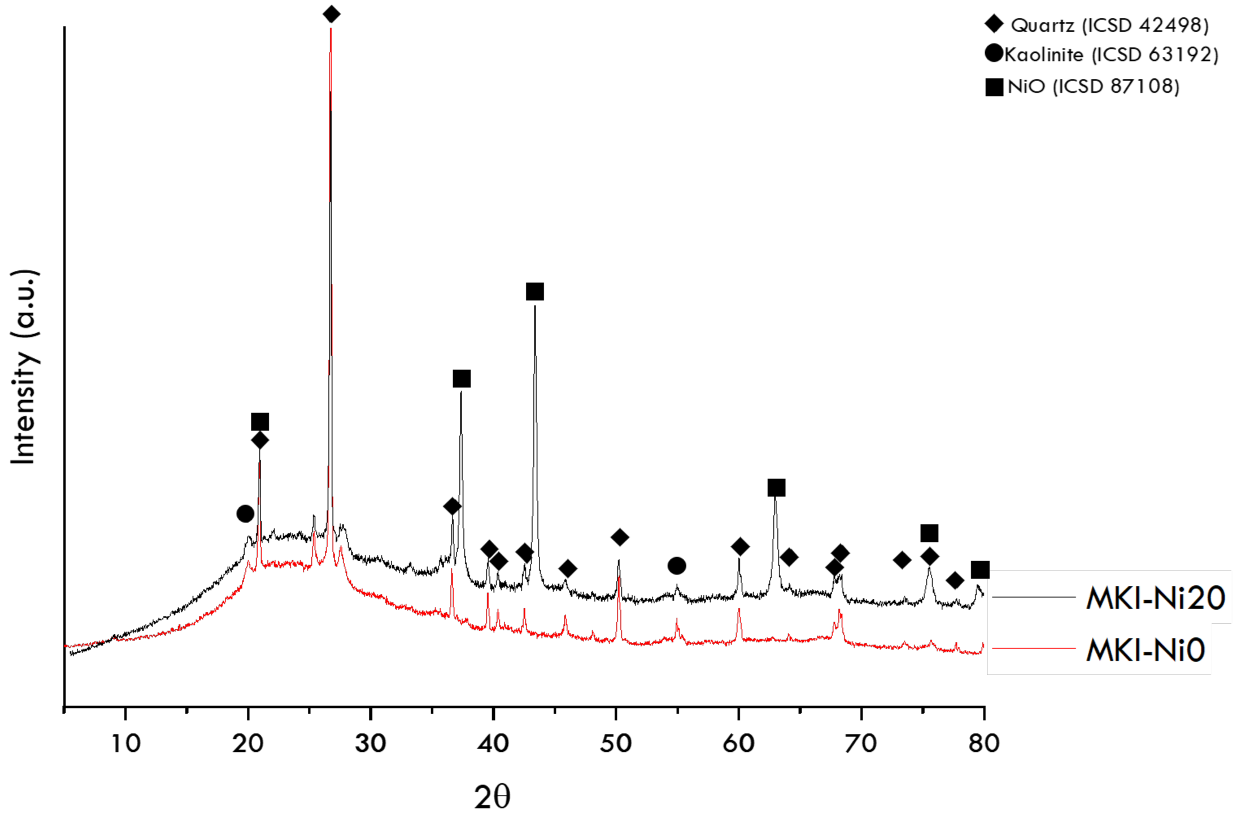

Figure S4: XRD profiles of MKI-Ni0 (800 °C calcined MK-I monolith) and MKI-Ni20 (800 °C calcinated Ni-impregnated MKI-Ni0 monolith
